# Impact of diabetes on remodelling, microvascular function and exercise capacity in aortic stenosis

**DOI:** 10.1136/openhrt-2023-002441

**Published:** 2023-08-16

**Authors:** Abhishek Dattani, Emer M Brady, Aseel Alfuhied, Gaurav S Gulsin, Christopher D Steadman, Jian L Yeo, Saadia Aslam, Marko Banovic, Michael Jerosch-Herold, Hui Xue, Peter Kellman, Philippe Costet, Mary Ellen Cvijic, Lei Zhao, Christina Ebert, Laura Liu, Kushan Gunawardhana, David Gordon, Ching-Pin Chang, J Ranjit Arnold, Thomas Yates, Damian Kelly, Kai Hogrefe, Dana Dawson, John Greenwood, Leong L Ng, Anvesha Singh, Gerry P McCann

**Affiliations:** 1 Department of Cardiovascular Sciences and NIHR Leicester Biomedical Research Centre, University of Leicester, Leicester, UK; 2 Department of Cardiology, Poole Hospital NHS Foundation Trust, Poole, UK; 3 Cardiology Department, Clinical Centre of Serbia, Belgrade, Serbia; 4 Department of Radiology, Brigham and Women's Hospital, Boston, Massachusetts, USA; 5 National Heart, Lung and Blood Institute, National Institutes of Health, Bethesda, Maryland, USA; 6 Bristol Myers Squibb Co, Princeton, New Jersey, USA; 7 Diabetes Research Centre, University of Leicester, Leicester, UK; 8 Cardiology Department, Royal Derby Hospital, Derby, UK; 9 Cardiology Department, Kettering General Hospital NHS Foundation Trust, Kettering, UK; 10 Cardiovascular Medicine Research Unit, University of Aberdeen, Aberdeen, UK; 11 Department of Cardiology, Leeds Teaching Hospitals NHS Trust, Leeds, UK; 12 Department of Biomedical Imaging Sciences, University of Leeds, Leeds, UK

**Keywords:** aortic valve stenosis, diabetes mellitus, magnetic resonance imaging

## Abstract

**Objective:**

To characterise cardiac remodelling, exercise capacity and fibroinflammatory biomarkers in patients with aortic stenosis (AS) with and without diabetes, and assess the impact of diabetes on outcomes.

**Methods:**

Patients with moderate or severe AS with and without diabetes underwent echocardiography, stress cardiovascular magnetic resonance (CMR), cardiopulmonary exercise testing and plasma biomarker analysis. Primary endpoint for survival analysis was a composite of cardiovascular mortality, myocardial infarction, hospitalisation with heart failure, syncope or arrhythmia. Secondary endpoint was all-cause death.

**Results:**

Diabetes (n=56) and non-diabetes groups (n=198) were well matched for age, sex, ethnicity, blood pressure and severity of AS. The diabetes group had higher body mass index, lower estimated glomerular filtration rate and higher rates of hypertension, hyperlipidaemia and symptoms of AS. Biventricular volumes and systolic function were similar, but the diabetes group had higher extracellular volume fraction (25.9%±3.1% vs 24.8%±2.4%, p=0.020), lower myocardial perfusion reserve (2.02±0.75 vs 2.34±0.68, p=0.046) and lower percentage predicted peak oxygen consumption (68%±21% vs 77%±17%, p=0.002) compared with the non-diabetes group. Higher levels of renin (log_10_renin: 3.27±0.59 vs 2.82±0.69 pg/mL, p<0.001) were found in diabetes. Multivariable Cox regression analysis showed diabetes was not associated with cardiovascular outcomes, but was independently associated with all-cause mortality (HR 2.04, 95% CI 1.05 to 4.00; p=0.037).

**Conclusions:**

In patients with moderate-to-severe AS, diabetes is associated with reduced exercise capacity, increased diffuse myocardial fibrosis and microvascular dysfunction, but not cardiovascular events despite a small increase in mortality.

**WHAT IS ALREADY KNOWN ON THIS TOPIC**

- Coexisting diabetes has a detrimental impact on outcomes in patients with aortic stenosis (AS), but detailed phenotyping of diabetes in AS versus AS alone has not been well described.

**WHAT THIS STUDY ADDS**

- Diabetes was associated with greater diffuse myocardial fibrosis, worse microvascular function, poorer exercise capacity and higher renin levels. Over a median follow-up of 6.9 years, multivariable analysis showed diabetes was not associated with increased cardiovascular events.

**HOW THIS STUDY MIGHT AFFECT RESEARCH, PRACTICE OR POLICY**

- Further large-scale studies could help better understand the impact of these distinct differences seen in AS patients with coexisting diabetes and assess the excess mortality seen in these patients.

## Introduction

Aortic stenosis (AS) is the most common valve disease requiring treatment in the developed world. Severe AS causes cardiac remodelling which leads to diastolic dysfunction, myocardial fibrosis and microvascular dysfunction, with poor prognosis without intervention.^1^

Diabetes is more common in patients with AS, and AS occurs more frequently in people with diabetes. Coexisting diabetes accelerates the progression of AS and is a determinant of poor outcomes in these patients.^1^ Despite this link, there is a poor understanding of the cardiovascular impact of diabetes in patients with AS.

Cardiovascular magnetic resonance imaging (CMR) can assess early changes in AS and diabetes. Cardiopulmonary exercise testing (CPET) provides important prognostic information in heart failure and exercise testing is recommended to guide treatment decisions in asymptomatic severe AS.^2^ Adverse profiles of plasma fibroinflammatory biomarkers have been shown in a range of conditions including AS and diabetes,^3 4^ but their role in patients with AS who have diabetes remains poorly characterised. The multiparametric capabilities of CMR together with CPET and fibroinflammatory biomarkers could yield new insights into the impact of diabetes in AS.

In this study, we sought to: (1) determine the differences in cardiac remodelling, exercise capacity and cardiovascular fibroinflammatory biomarkers in patients with moderate-to-severe AS with and without diabetes and (2) confirm the impact of diabetes on outcomes in AS. We hypothesised that AS patients with diabetes will have more adverse cardiac remodelling and worse clinical outcomes compared with those without diabetes.

## Methods

### Study design and participants

This is a secondary analysis of multicentre data pooled from three observational studies in the UK (two previously published^5 6^ and one ongoing: NCT03883490). Adults with either moderate or severe AS (two or more of: aortic valve area <1.5 cm^2^, peak velocity >3.0 m/s or mean pressure gradient >25 mm Hg) were prospectively recruited between 2009 and 2021. Exclusion criteria included other severe valve disease, atrial fibrillation or other significant arrhythmia, any contraindication to CMR or an estimated glomerular filtration rate (eGFR)<30 mL/min/1.73 m^2^. The core dataset included a transthoracic echocardiogram (TTE) and contrast-enhanced CMR such that all participants included in this analysis had these investigations performed. All participants provided written informed consent. Diabetes status was defined by a known history or a glycated haemoglobin (HbA1c) ≥6.5%.

### Blood sampling

Blood sampling was performed at time of recruitment and included haematocrit, renal function, high-sensitivity troponin I and N-terminal pro B-type natriuretic peptide. A subset of asymptomatic participants with moderate or severe AS had plasma stored for the quantification of circulating biomarkers, which was performed as a batch analysis at the end of the study using a bead-based multiplex assay on a Luminex platform (Bristol Myers Squibb, New Jersey, USA) as previously described.^7^ This consisted of 49 biomarkers known to be associated with myocardial injury and hypertrophy, fibrosis, atrial stretch, inflammation, oxidative stress, and renal and endothelial dysfunction (online supplemental table 1).

10.1136/openhrt-2023-002441.supp1 Supplementary data

### Cardiopulmonary exercise testing

Exercise capacity was assessed using an incremental symptom-limited CPET using a bicycle ergometer with a 1 min ramp protocol as previously described.^5^ Percentage predicted peak oxygen consumption (VO_2_) was calculated using the Wasserman/Hansen equation.

### Transthoracic echocardiography

TTE was performed with a Vivid 7 (GE Healthcare, Waukesha, Wisconsin) or iE33b system (Phillips Medical Systems, Best, Netherlands) using a standardised protocol. Image acquisition and reporting was undertaken as per the American Society of Echocardiography guidelines.

### Cardiovascular MR

Participants underwent adenosine-stress CMR imaging using either a 1.5-Tesla or 3-Tesla platform as previously described.^5 6^ In brief, long-axis and short-axis cine imaging of the whole heart was obtained using a balanced steady state free precession technique and retrospective electrocardiographic gating. Perfusion imaging was performed for three slices (basal, mid and apical) at rest and during pharmacological stress using 140–210 µg/kg/min of intravenous adenosine infused for 3–5 min and a gadolinium-based contrast agent for which either Magnevist (Bayer Healthcare, Germany), Gadovist (Bayer Pharma AG, Germany) or Dotarem (Guerbet, France) was used. Late gadolinium enhancement (LGE) imaging was performed using a segmented approach at least 10 min following final contrast injection. T1 mapping was available in a subset of participants, all of which were scanned at 3-Tesla, for whom a precontrast and postcontrast T1 map was performed at the mid-left ventricular (LV) level using a modified inversion recovery Look-Locker technique.

### Image analysis

All image analysis was performed at a core lab in Leicester, UK with blinding to participant details. TTE images were analysed by an accredited cardiac sonographer using an Xcelera (Philips, Best, The Netherlands) workstation. Diastolic dysfunction grading was assessed using international recommendations.^8^

CMR image quantitative analysis was performed in batch analysis by a single observer (AD) using cvi42 (V. 5.10.1, Circle Cardiovascular Imaging, Calgary, Canada) with methods detailed in online supplemental material.

### Clinical follow-up

Outcome data were obtained from electronic health records with blinding to diabetes status and a minimum follow-up of 1 year. The primary endpoint was a composite of cardiovascular death (as defined by diagnosis on death certificate), myocardial infarction, hospitalisation with heart failure (requiring intravenous treatment), syncope or any significant arrhythmia (including significant atrioventricular block or tachyarrhythmia), with outcome definitions as previously described in the literature.^9^ A secondary endpoint of all-cause mortality was also assessed.

### Statistical analysis

Baseline characteristics were compared using an independent t-test, Mann-Whitney U test or χ^2^ test as appropriate. Imaging parameters were compared between groups using analysis of covariance (ANCOVA) with age, sex, ethnicity, systolic blood pressure, eGFR, body mass index and aortic valve mean pressure gradient as covariates. The key outcome from CPET was percentage predicted peak VO_2_ and, therefore, comparison of CPET variables was adjusted for systolic blood pressure, eGFR and aortic valve mean pressure gradient. A sensitivity analysis excluding participants with HbA1c values within the pre-diabetes range (6.0%–6.4%) was also performed. Plasma biomarker data were initially cleaned and treated as either continuous variables or dichotomised into ‘high’ and ‘low’ where appropriate as detailed in online supplemental material. Between-group comparison of plasma biomarkers was corrected for multiple testing using the Benjamini-Hochberg method with a false discovery rate of 0.05.^10^

### Clinical outcome data

Kaplan-Meier curves were generated and log-rank test and HRs were used to assess differences in outcomes by diabetes status. Given the importance of aortic valve replacement, separate analyses were also performed using Kaplan-Meier curves stratified by the presence and absence of valve replacement. Cox regression with key clinical characteristics (age, aortic valve replacement, aortic valve mean pressure gradient and diabetes status) was conducted to produce a baseline model with further models generated using key imaging parameters and fibroinflammatory markers.

A p<0.05 was considered statistically significant throughout. Descriptive statistics, ANCOVA and survival curves were performed using SPSS Statistics (V.28.0, IBM). Graphs were generated using GraphPad Prism (V.9.0.0, San Diego, California, USA).

### Patient and public involvement

Patient and public involvement was undertaken during the design of the original studies which have been used as part of this study. Lay members of the public were part of study steering committees.

## Results

### Baseline characteristics

**Figure 1:**
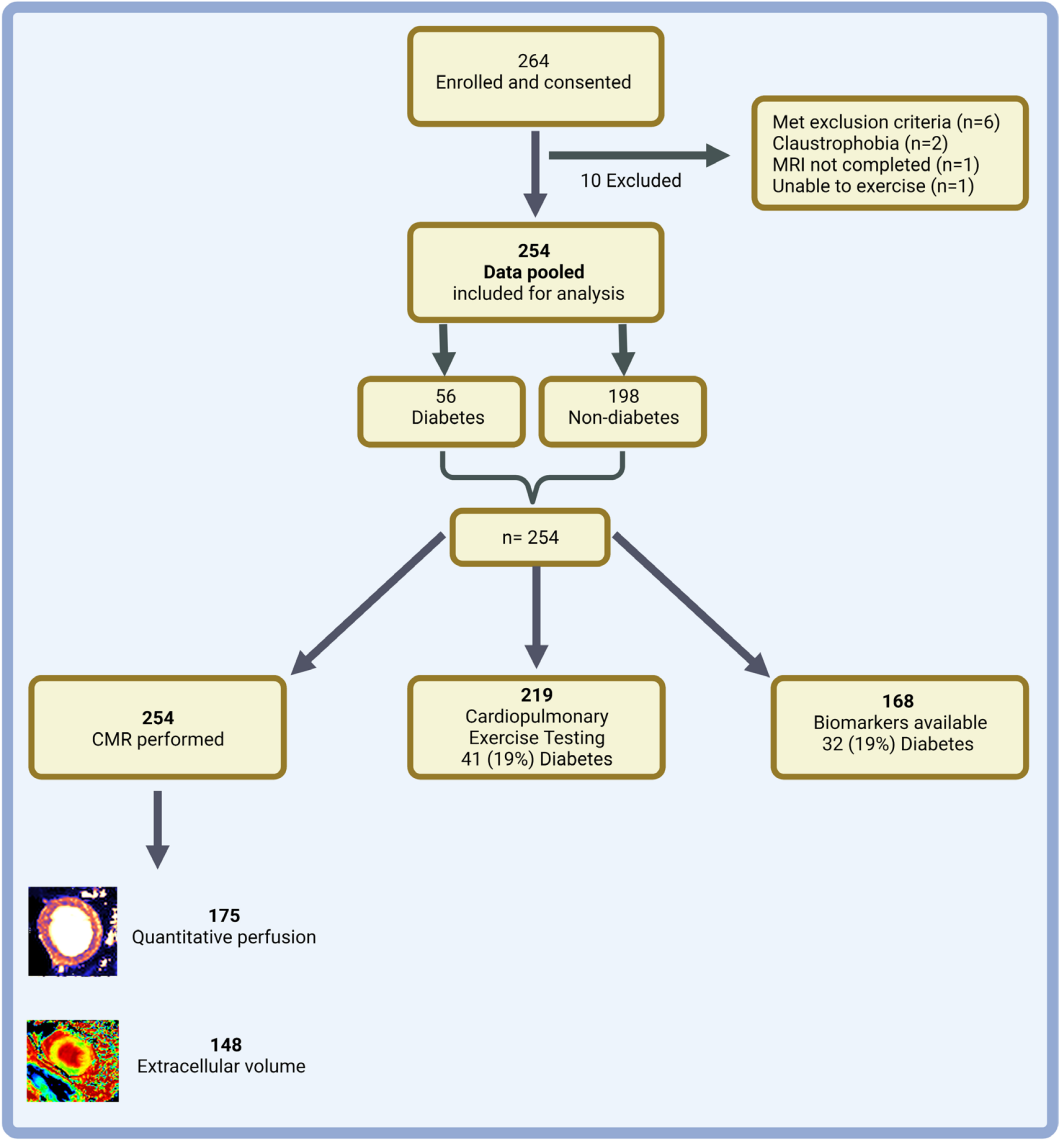
Study flow diagram. Summary of study enrolment, exclusions and number of participants in each group. Created with BioRender.com. CMR, cardiovascular MRI.

A total of 254 participants (figure 1 and online supplemental figure 1) were included in this analysis and stratified into two groups according to diabetes status: diabetes (n=56) and non-diabetes (n=198). Baseline characteristics are presented in table 1. The groups were well matched for age, sex, ethnicity, blood pressure and severity of AS. The diabetes group had higher body mass index and HbA1c level, lower eGFR and were more likely to have a history of hypertension, hyperlipidaemia and symptoms of AS.

**Table 1:** Baseline characteristics comparing aortic stenosis participants with and without diabetes

	Diabetes (n=56)	Non-diabetes (n=198)	P value
Age	70 (63–75)	69 (61–75)	0.361
Sex, n (%) male	44 (79)	149 (75)	0.608
Ethnicity, n (%) white	53 (95)	195 (99)	0.095
Height, m	1.68±0.10	1.70±0.09	0.121
Weight, kg	85.3±17.4	81.8±14.7	0.133
Body mass index, kg/m^2^	30.1±5.2	28.0±4.2	0.003
Systolic blood pressure, mm Hg	146±21	142±22	0.287
Diastolic blood pressure, mm Hg	75±12	77±10	0.146
Heart rate, bpm	72±14	69±11	0.128
HbA1c, mmol/mol	51±11	38±4	<0.001
HbA1c, %	6.8±1.0	5.7±0.4	<0.001
Medical history			
Hypertension, n (%)	45 (80)	101 (51)	<0.001
Hyperlipidaemia, n (%)	35 (63)	85 (43)	0.010
Smoking history, n (%)	33 (59)	110 (56)	0.653
Ischaemic heart disease, n (%)	26 (46)	77 (39)	0.310
Severe AS, n (%)	48 (86)	155 (78)	0.220
Symptomatic AS, n (%)	22 (39)	50 (25)	0.040
Medications			
ACEi/ARB, n (%)	38 (68)	69 (35)	<0.001
Beta blocker, n (%)	24 (43)	64 (33)	0.158
Diuretic, n (%)	24 (43)	47 (24)	0.006
Statin, n (%)	50 (89)	109 (56)	<0.001
Bloods and valve severity			
eGFR, mL/min/1.73 m^2^	71 (60–87)	81 (66–96)	0.022
NTproBNP, pmol/L	104 (39–257)	73 (21–226)	0.121
hsTnI, pg/mL	6.4 (4.1–10.7)	5.5 (3.3–10.1)	0.414
AV peak velocity, m/s	4.0±0.6	4.0±0.6	0.732
AV maximum pressure gradient, mm Hg	67±20	65±21	0.696
AV mean pressure gradient, mm Hg	38±12	39±14	0.936
AV area, cm^2^	0.96±0.34	1.05±0.30	0.055

Data presented as mean (SD), median (IQR) or number (%) as appropriate.

ACEi, ACE inhibitor; ARB, angiotensin receptor blocker; AS, aortic stenosis; AV, aortic valve; eGFR, estimated glomerular filtration rate; HbA1c, glycated haemoglobin; hsTnI, High sensitivity troponin I; NTproBNP, N-terminal pro-B-type natriuretic peptide.

### Imaging data

Key imaging data are presented in table 2. The diabetes group had a higher E/A ratio compared with non-diabetes but e’ and E:e’ ratio were similar between groups. There was no significant difference in diastolic function grading between the diabetes and non-diabetes groups.

**Table 2:** Comparison of imaging and exercise testing between diabetes and non-diabetes groups

	Diabetes (n=56)	Non-diabetes (n=198)	P value
Echocardiography*			
E/A ratio	0.91±0.36	0.87±0.28	0.047
Septal e’ (cm/s)	6.8±4.2	6.4±3.4	0.090
Lateral e’ (cm/s)	7.7±3.2	7.9±2.9	0.282
E:e’ ratio	13.2±5.6	11.3±4.4	0.176
Diastolic function, n (%)			0.169†
Normal	32 (57)	134 (68)	
Grade I/indeterminate	22 (39)	57 (29)	
Grade II/III	2 (4)	7 (4)	
CMR*			
LV EDVi (mL/m)	88±25	90±21	0.316
LV ESVi (mL/m)	29±17	27±11	0.451
LV EF (%)	68±11	70±7	0.129
LVMi (g/m)	97±22	95±24	0.658
LVM/EDV (g/mL)	1.14±0.24	1.07±0.22	0.226
LV GCS (%)	17.0±3.4	18.2±3.1	0.090
LV GLS (%)	14.0±3.5	14.5±2.7	0.758
LV circumferential PEDSR (s^-1^)	0.67±0.19	0.71±0.24	0.999
LV longitudinal PEDSR (s^-1^)	0.53±0.21	0.54±0.18	0.176
RV EDVi (mL/m)	94±26	99±18	0.090
RV ESVi (mL/m)	41±15	43±12	0.399
RV EF (%)	57±7	57±7	0.559
Maximum LAVi (mL/m)	43±14	43±16	0.303
Maximum LAV/EDV ratio	0.50±0.14	0.49±0.16	0.809
LA EF (%)	55±13	57±11	0.981
Presence of LGE, n (%)	32 (59)	102 (52)	0.346†
Non-ischaemic	23 (43)	80 (41)	0.814†
Infarction	9 (17)	21 (11)	0.233†
Native T1 (ms)	1172±83	1149±75	0.881
Extracellular volume (%)	25.9±3.1	24.8±2.4	0.020
Myocardial perfusion reserve	2.02±0.75	2.34±0.68	0.046
CPET‡			
CPET duration (s)	485±127	535±135	0.050
Peak load (watts)	90±34	110±40	0.006
Peak VO_2_ (mL/kg/min)	14.7±4.9	17.4±5.2	0.006
Percentage predicted peak VO_2_ (%)	68±21	77±17	0.002
Peak respiratory exchange ratio	1.12±0.16	1.11±0.13	0.494
Peak heart rate	127±17	134±21	0.107
Percentage predicted heart rate (%)	84±10	87±12	0.152
Peak O_2_ pulse	9.6±3.4	10.7±3.0	0.075

Ventricular volumes and mass were indexed to height. Predicted peak VO2 calculated using the Wasserman/Hansen equation. Values presented as mean (SD) or n (%) as appropriate. CPET was performed on 219 participants (41 diabetes, 178 non-diabetes).

*ANCOVA adjusted for age, sex, ethnicity, systolic BP, eGFR, BMI and aortic valve mean pressure gradient.

†χ^2^ test.

‡ANCOVA adjusted for systolic BP, eGFR and aortic valve mean pressure gradient.

ANCOVA, analysis of covariance; BMI, body mass index; BP, blood pressure; CMR, cardiovascular MR; CPET, cardiopulmonary exercise testing; EDVi, indexed end-diastolic volume; EF, ejection fraction; eGFR, estimated glomerular filtration rate; ESVi, indexed end-systolic volume; GCS, global circumferential strain; GLS, global longitudinal strain; LAVi, indexed left atrial volume; LGE, late gadolinium enhancement; LV, left ventricle; LVMi, indexed left ventricular mass; PEDSR, peak early diastolic strain rate; RV, right ventricular; VO2, oxygen consumption.

On CMR, LV volumes were similar between the groups. There was no difference in LV mass, systolic and diastolic function or patterns of LGE between the groups. Extracellular volume fraction (ECV) was available in 148 participants (33 diabetes and 115 non-diabetes) and showed that the diabetes group had more diffuse fibrosis (ECV: 25.9%±3.1% vs 24.8%±2.4%, p=0.020). After exclusions, myocardial perfusion reserve (MPR) was assessed in 175 participants (31 diabetes, 144 non-diabetes) and demonstrated worse microvascular function (MPR: 2.02±0.75 vs 2.34±0.68, p=0.046) in the diabetes group compared with the non-diabetes group. Consistent findings in key imaging variables were demonstrated during sensitivity analysis with exclusion of participants with pre-diabetes (online supplemental table 2), although differences in ECV no longer reached statistical significance.

### CPET data

CPET was undertaken in 219 participants (table 2). Patients with diabetes had lower percentage predicted peak VO_2_ (68%±21% vs 77%±17%, p=0.002), peak work load (90±34 vs 110±40 watts, p=0.006) and exercise duration (8.1±2.1 vs 8.9±2.3 min, p=0.050) compared with non-diabetes. Sensitivity analysis excluding participants with pre-diabetes showed consistent findings (online supplemental table 2).

### Biomarkers

**Figure 2:**
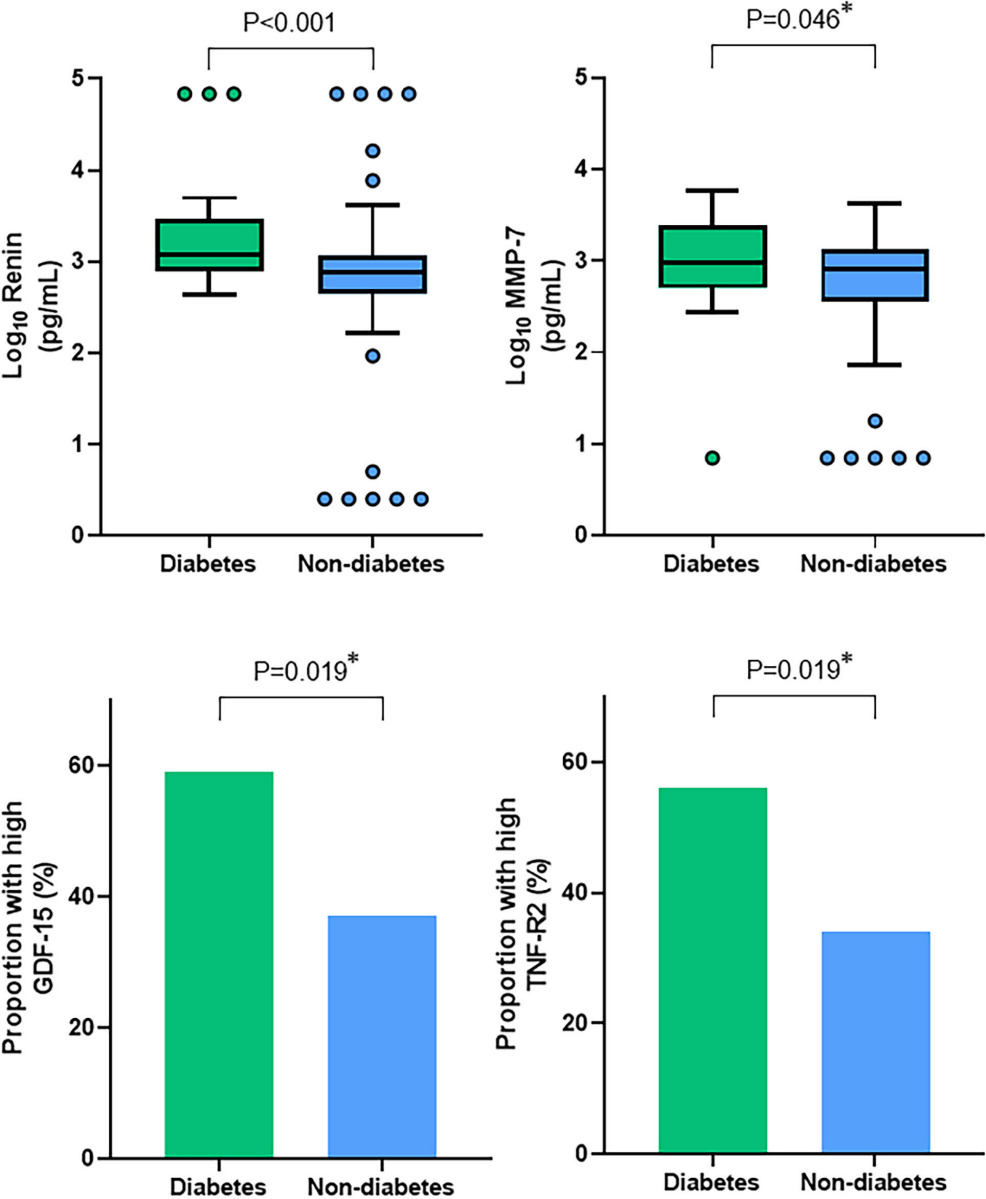
Key differences in biomarkers between the diabetes and non-diabetes groups. Diabetes was associated with higher levels of Renin and MMP-7 compared with the non-diabetes group, and a greater proportion of patients with diabetes had high levels of GDF-15 and TNF-R2. *Not significantly different following correction for multiple testing. GDF-15, growth differentiation factor-15, MMP-7, matrix metalloproteinase-7, TNF-R2, tumour necrosis factor-receptor 2.

Higher levels of matrix metalloproteinase-7 (log_10_MMP-7: 3.00±0.55 vs 2.78±0.55 pg/mL, p=0.046) and renin (log_10_renin: 3.27±0.59 vs 2.82±0.69 pg/mL, p<0.001), and greater proportion of patients with high levels of growth differentiation factor-15 (59% vs 37%, p=0.019) and tumour necrosis factor-receptor 2 (56% vs 34%, p=0.019) were seen in patients with diabetes compared with those without (figure 2, online supplemental tables 3 and 4). On correction for multiple comparisons, only renin remained significantly different between the two groups.

### Outcomes

**Figure 3:**
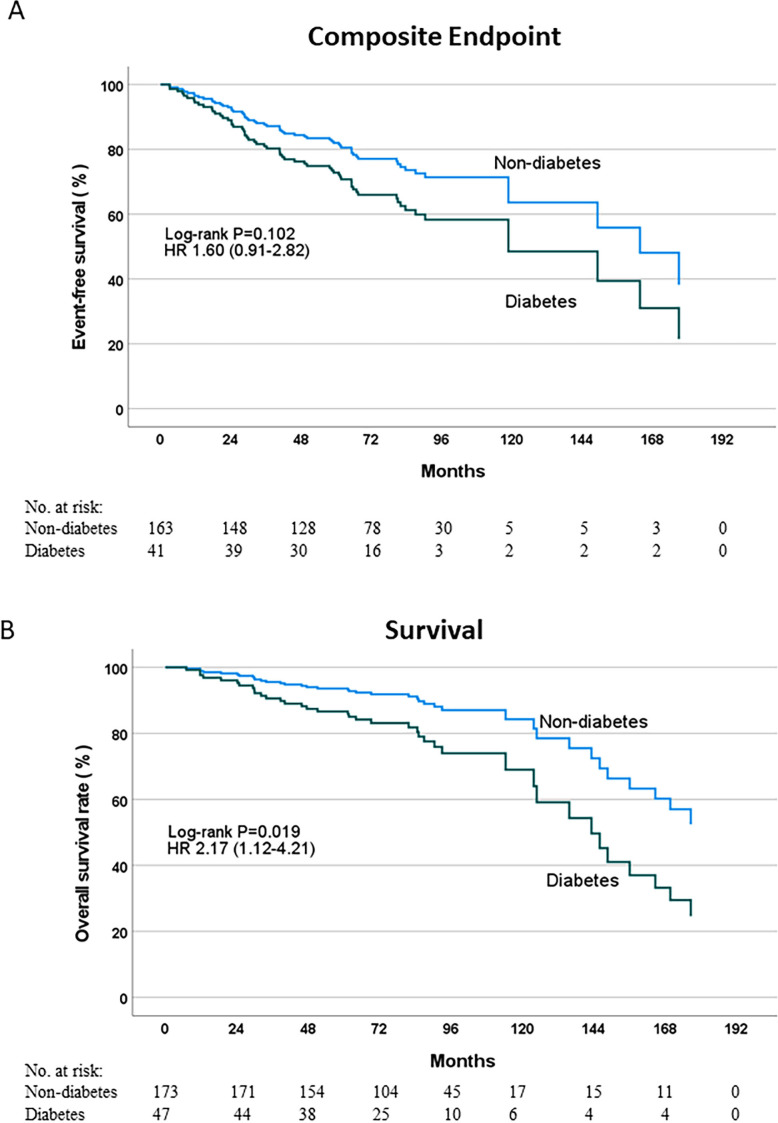
Outcome analysis. Kaplan-Meier curves showing event-free survival to a composite endpoint of cardiovascular death, heart failure hospitalisation, myocardial infarction, syncope or arrythmia (A) and overall survival (B) between patients with and without diabetes.

Aortic valve replacement was undertaken in 89% of the diabetes group and 83% of the non-diabetes group. Composite outcome data were available in 204 (80%) participants. Over a median follow-up of 6.9 years (range 1–15.7 years), 59 (29%) participants reached the primary composite endpoint comprising 17 (41%) of the diabetes group compared with 42 (26%) of the non-diabetes group (log-rank p=0.102; HR 1.60; 95% CI 0.91 to 2.82; figure 3). On assessment of participants stratified by the presence (composite endpoint reached: 16 (46%) diabetes, 36 (29%) non-diabetes) or absence (composite endpoint reached: 1 (17%) diabetes, 6 (16%) non-diabetes) of aortic valve replacement, similar findings of no significant difference were shown between those with diabetes compared with those without diabetes (with valve replacement: log-rank p=0.117, HR 1.60, 95% CI 0.89 to 2.90; without valve replacement: log-rank p=0.999, HR 0.998, 95% CI 0.120 to 8.305).

Cox regression model analysis demonstrated diabetes was not independently associated with this composite outcome whereas age and the presence of LGE and aortic valve replacement were associated factors (table 3). The addition of MPR or ECV did not improve the model and neither did the addition of key fibroinflammatory markers (online supplemental table 5).

**Table 3:** Cox regression models for primary composite endpoint and secondary endpoint

	Primary composite endpoint								Mortality endpoint			
	Baseline model (n=204)		Baseline+LGE (n=201)		Baseline+MPR (n=143)		Baseline+ECV (n=129)		Baseline (n=220)		Baseline+LGE (n=217)	
	HR (95% CI)	P value	HR (95% CI)	P value	HR (95% CI)	P value	HR (95% CI)	P value	HR (95% CI)	P value	HR (95% CI)	P value
Age	1.09 (1.05 to 1.12)	<0.001	1.08 (1.05 to 1.11)	<0.001	1.09 (1.04 to 1.13)	<0.001	1.08 (1.03 to 1.12)	<0.001	1.04 (1.00 to 1.07)	0.052	1.03 (0.98 to 1.07)	0.091
Diabetes	1.34 (0.76 to 2.37)	0.316	1.29 (0.72 to 2.30)	0.395	1.75 (0.83 to 3.69)	0.142	1.21 (0.55 to 2.65)	0.636	2.04 (1.05 to 4.00)	0.037	2.15 (1.10 to 4.20)	0.026
AV mean PG	0.98 (0.97 to 1.00)	0.089	0.98 (0.96 to 1.00)	0.060	0.99 (0.97 to 1.02)	0.604	0.99 (0.96 to 1.02)	0.445	1.01 (0.99 to 1.04)	0.265	1.01 (0.99 to 1.04)	0.324
AVR	2.45 (1.09 to 5.51)	0.031	3.08 (1.28 to 7.38)	0.012	2.55 (0.86 to 7.58)	0.093	2.27 (0.79 to 6.54)	0.129	0.27 (0.11 to 0.63)	0.002	0.31 (0.13 to 0.75)	0.010
LGE	NA	NA	1.88 (1.08 to 3.30)	0.027	NA	NA	NA	NA	NA	NA	1.90 (0.94 to 3.87)	0.075
MPR	NA	NA	NA	NA	1.12 (0.63 to 1.99)	0.710	NA	NA	NA	NA	NA	NA
ECV	NA	NA	NA	NA	NA	NA	1.02 (0.88 to 1.17)	0.814	NA	NA	NA	NA

AVR, aortic valve replacement; PG, pressure gradient; LGE, late gadolinium enhancement; MPR, myocardial perfusion reserve; ECV, extracellular volume fraction; NA, not applicable.

Data for the secondary outcome were available in 220 participants. Thirty-eight (17%) participants died, comprising 14 (30%) of the diabetes group and 24 (14%) of the non-diabetes group (log-rank p=0.019, HR 2.17, 95% CI 1.12 to 4.21; figure 3). Stratification based on the presence (secondary endpoint reached: 11 (27%) diabetes, 17 (13%) non-diabetes) or absence (secondary endpoint reached: 3 (50%) diabetes, 7 (19%) non-diabetes) of valve replacement demonstrated similar findings in those with valve replacement (log-rank p=0.037, HR 2.21, 95% CI 1.03 to 4.72) but this did not reach significance in those without valve replacement (log-rank p=0.098, HR 2.98, 95% CI 0.77 to 11.59).

Cox regression model analysis demonstrated that diabetes was independently associated with all-cause mortality (HR 2.04, 95% CI 1.05 to 4.00, p=0.037; table 3).

Sensitivity analysis excluding participants with pre-diabetes demonstrated consistent findings of no difference in composite endpoint (log-rank p=0.058, HR 1.76, 95% CI 0.97 to 3.18) between the diabetes and non-diabetes groups, but the diabetes group were more likely to reach the secondary endpoint of all-cause death (log-rank p=0.029, HR 2.08, 95% CI 1.06 to 4.07).

## Discussion

In this cohort of moderate-severe AS patients who underwent extensive phenotyping, we have demonstrated unique cardiovascular effects of diabetes in AS. Despite having similar AS severity, those with diabetes had more diffuse fibrosis, worse microvascular function and poorer exercise capacity.

To our knowledge, only one other study has directly evaluated the impact of diabetes on CV structure/function in AS. Lee *et al*^11^ showed worse diastolic function and more diffuse interstitial fibrosis in their diabetes group compared with non-diabetes. They also demonstrated an elevation of a range of biomarkers involved with inflammation and the modulation of extracellular matrix in diabetes. A significant limitation was that study groups were not age matched and there were two separate cohorts for imaging and biomarkers. Furthermore, their study lacked stress-perfusion CMR or CPET for which we have been able to demonstrate novel important differences.

### Fibrosis, microvascular function and cardiac remodelling

ECV is a surrogate for diffuse interstitial fibrosis. In diabetes alone, ECV has been shown to be higher compared with controls and ECV is associated with heart failure hospitalisation and mortality.^12^ Indeed, ECV has been associated with markers of disease severity and all-cause mortality in patients with AS, even after aortic valve replacement.^13^ We found a small increase in diffuse fibrosis in the diabetes group with AS which is consistent with a previous study that examined preoperative LV biopsies, although in a much smaller sample size (n=16 with diabetes),^14^ as well as in the study by Lee *et al.*^11^ In diabetes, the mechanism of cardiac fibrosis is complex but may be related to changes in fibroinflammatory markers (such as transforming growth factor or matrix metalloproteinases)^15^ which may lead to the deposition of collagen within the extracellular matrix. This can cause increased myocardial stiffness which could help drive the myocardial hypertrophic response from being adaptive to decompensation^1^ and contribute to microvascular dysfunction. In our analysis, however, only renin remained significantly different between the groups once correction for multiple comparisons was performed. Renin is known to play a role in myocardial stiffness and contributes to cardiac fibrosis via the renin–angiotensin–aldosterone system.^16^ The lack of significant differences in other markers is surprising given the plethora of evidence suggesting increased fibroinflammatory markers in diabetes.^4^ The absence of a more adverse fibroinflammatory profile in this cohort may explain why a similar proportion of our diabetes group had LGE compared with the non-diabetes group even though diabetes is associated with focal cardiac fibrosis as detected by LGE.^17^

MPR is a non-invasive method of assessing coronary microvascular dysfunction and is impaired in diabetes.^18^ In patients without significant epicardial coronary disease, coronary microvascular dysfunction is associated with diastolic dysfunction and heart failure hospitalisation.^19^ In AS, MPR has been associated with onset of symptoms^6^ and ECV is independently associated with MPR.^20^ Our findings of more diffuse fibrosis and worse microvascular function in AS patients with diabetes compared with those without diabetes are in keeping with previous studies examining diabetes^12 18^ or AS.^5 21^ Importantly, diffuse fibrosis^22^ and microvascular dysfunction^23^ have been shown to be partially reversible with valve replacement but whether specific improvement of these parameters with targeted treatments leads to improved outcomes is unknown. MPR and ECV, however, did not help predict events in our cohort but this may be due to the fact that the vast majority of patients subsequently had aortic valve replacement.

Surprisingly, little difference was seen in other measures of cardiac remodelling such as LV volumes, mass, LV mass/volume ratio, LV strain as well as left atrial and right ventricular volumes. We have previously demonstrated that people with diabetes have smaller LV volumes compared with controls.^24^ This analysis, as well as in the study by Lee *et al*, has shown similar LV volumes in AS patients with diabetes compared with those without diabetes. This surprising result may be because AS can lead to increased LV wall tension and a subsequent dilatation of the LV^25^ and thus counteract against the smaller LV cavity size often seen in diabetes. We saw minimal changes in diastolic function which may be due to similar age and severity of AS between groups.

### Exercise capacity

The coexistence of diabetes and AS was associated with significant impairment of exercise capacity compared with the non-diabetes group. Both groups had a mean percentage predicted peak VO_2_ below the 85% reference range^26^ which is in agreement with previously known reduced exercise capacity in AS^27^ and in diabetes^28^ alone. However, given that peak VO_2_ is independently associated with survival in patients with AS,^27^ our finding of a 12% lower mean percentage predicted peak VO_2_ in the diabetes group compared with the non-diabetes group is a key novel finding from this study and may reflect on the multisystemic impact of diabetes.

This reduction in exercise performance may be related to a range of biological alterations seen in diabetes such as disturbances in endothelium-mediated vasodilation which is responsible for exercise hyperaemia, microangiopathy leading to skeletal muscle dysfunction or autonomic dysfunction resulting in an impaired chronotropic response during exercise.^29^ Peak heart rate, however, was not significantly different between our groups making the latter mechanism less likely in this cohort. MPR has been shown to be associated with exercise capacity in patients with diabetes^18^ and in patients with AS^5 20^ and may also partly explain the reduced exercise capacity seen in our diabetes group.

### Outcomes in diabetes

Our data have shown that diabetes is not an independent predictor of cardiovascular mortality and cardiac events. However, these patients clearly do die earlier and our secondary outcome data showed that diabetes remains independently associated with all-cause mortality. That LGE was a significant factor in cardiovascular outcomes is not surprising and is consistent with previous findings.^30^ The fact that valve replacement was positively associated with cardiac outcomes is likely because a large proportion of our patients would have developed symptoms such as syncope and heart failure, which were part of our composite endpoint, and therefore went on to have a valve replacement. Indeed, valve replacement had a significant protective effect on all-cause death.

With a similar length of follow-up (median 6.3 years) compared with our study, Lee *et al* showed diabetes is significantly associated with outcomes (HR 1.88).^11^ Their events, however, were largely driven by all-cause death which would be somewhat consistent with our secondary outcome findings. Other groups have shown that diabetes increases the risk of non-cardiac deaths.^31^ Our findings, and those from other groups, show that diabetes does lead to a poorer prognosis in AS but this is likely driven by non-cardiovascular complications of diabetes, rather than due to the small impact we have seen on diffuse fibrosis and microvascular function.

### Strengths and limitations

To our knowledge, this is the only study to assess the impact of diabetes on AS with the depth of phenotypic detail covering multimodality imaging together with CPET and plasma biomarkers in a single cohort. A limitation is the smaller number of participants undergoing CPET, plasma biomarkers, quantitative perfusion and ECV, but these data still represent one of the most comprehensive descriptions of this cohort in the literature. The data presented are a retrospective analysis of prospectively collected data for which the primary aim was not to evaluate the differences between the presence or absence of diabetes. Although the analysis was blinded to diabetes status, the data should be viewed as exploratory and hypothesis-generating.

### Conclusion

In patients with moderate-severe AS, diabetes was associated with a reduction in exercise capacity and perfusion reserve and a small increase in diffuse fibrosis, but no increase in myocardial scar and minimal evidence of circulating fibroinflammatory biomarker activation. Furthermore, diabetes was not associated with increased cardiovascular events during a median follow-up of 6.9 years, despite a small increase in mortality. Further large-scale research is needed to assess whether the excess mortality associated with diabetes in patients with AS is primarily related to non-cardiovascular deaths.

## Data Availability

Data are available on reasonable request. The datasets used in the current study are available from the corresponding author on reasonable request.

## References

[1] Banovic M, Athithan L, McCann GP. Aortic stenosis and diabetes mellitus: an ominous combination. Diab Vasc Dis Res 2019;16:310–23. 10.1177/1479164118820657 30623669

[2] Otto CM, Nishimura RA, Bonow RO, et al. ACC/AHA guideline for the management of patients with valvular heart disease: A report of the American college of cardiology/American heart Association joint committee on clinical practice guidelines. Circulation 2021;143:e72–227. 10.1161/CIR.0000000000000923 33332150

[3] Lindman BR, Breyley JG, Schilling JD, et al. Prognostic utility of novel biomarkers of cardiovascular stress in patients with aortic stenosis undergoing valve replacement. Heart 2015;101:1382–8. 10.1136/heartjnl-2015-307742 26037104 PMC4598053

[4] Gulsin GS, Kanagala P, Chan DCS, et al. Differential left ventricular and left atrial remodelling in heart failure with preserved ejection fraction patients with and without diabetes. Ther Adv Endocrinol Metab 2019;10:2042018819861593. 10.1177/2042018819861593 31308926 PMC6613057

[5] Steadman CD, Jerosch-Herold M, Grundy B, et al. Determinants and functional significance of myocardial perfusion Reserve in severe aortic stenosis. JACC Cardiovasc Imaging 2012;5:182–9. 10.1016/j.jcmg.2011.09.022 22340825

[6] Singh A, Greenwood JP, Berry C, et al. Comparison of exercise testing and CMR measured myocardial perfusion reserve for predicting outcome in asymptomatic aortic stenosis: the Prognostic importance of Microvascular dysfunction in aortic stenosis (PRIMID AS) study. Eur Heart J 2017;38:1222–9. 10.1093/eurheartj/ehx001 28204448 PMC5837288

[7] Singh A, Chan DCS, Greenwood JP, et al. Symptom onset in aortic stenosis: relation to sex differences in left ventricular remodeling. JACC Cardiovasc Imaging 2019;12:96–105. 10.1016/j.jcmg.2017.09.019 29248646

[8] Nagueh SF, Smiseth OA, Appleton CP, et al. Recommendations for the evaluation of left ventricular diastolic function by echocardiography: an update from the American society of echocardiography and the European Association of cardiovascular imaging. J Am Soc Echocardiogr 2016;29:277–314. 10.1016/j.echo.2016.01.011 27037982

[9] Hicks KA, Mahaffey KW, Mehran R, et al. Cardiovascular and stroke Endpoint definitions for clinical trials. Circulation 2018;137:961–72. 10.1161/CIRCULATIONAHA.117.033502 29483172

[10] Benjamini Y, Hochberg Y. Controlling the false discovery rate: A practical and powerful approach to multiple testing. J Royal Statist Soc 1995;57:289–300. 10.1111/j.2517-6161.1995.tb02031.x Available: http://doi.wiley.com/10.1111/rssb.1995.57.issue-1

[11] Lee H-J, Park CS, Lee S, et al. Systemic Proinflammatory−Profibrotic response in aortic stenosis patients with diabetes and its relationship with myocardial remodeling and clinical outcome. Cardiovasc Diabetol 2023;22:30. 10.1186/s12933-023-01763-1 36765354 PMC9921197

[12] Wong TC, Piehler KM, Kang IA, et al. Myocardial extracellular volume fraction quantified by cardiovascular magnetic resonance is increased in diabetes and associated with mortality and incident heart failure admission. Eur Heart J 2014;35:657–64. 10.1093/eurheartj/eht193 23756336 PMC3945798

[13] Chin CWL, Everett RJ, Kwiecinski J, et al. Myocardial fibrosis and cardiac Decompensation in aortic stenosis. JACC Cardiovasc Imaging 2017;10:1320–33. 10.1016/j.jcmg.2016.10.007 28017384 PMC5683736

[14] Falcão-Pires I, Hamdani N, Borbély A, et al. Diabetes mellitus worsens diastolic left ventricular dysfunction in aortic stenosis through altered myocardial structure and cardiomyocyte stiffness. Circulation 2011;124:1151–9. 10.1161/CIRCULATIONAHA.111.025270 21844073

[15] Westermann D, Rutschow S, Jäger S, et al. Contributions of inflammation and cardiac matrix metalloproteinase activity to cardiac failure in diabetic cardiomyopathy: the role of angiotensin type 1 receptor antagonism. Diabetes 2007;56:641–6. 10.2337/db06-1163 17327431

[16] Jia G, Aroor AR, Hill MA, et al. Role of renin-angiotensin-aldosterone system activation in promoting cardiovascular fibrosis and stiffness. Hypertension 2018;72:537–48. 10.1161/HYPERTENSIONAHA.118.11065 29987104 PMC6202147

[17] Bojer AS, Sørensen MH, Vejlstrup N, et al. Distinct non-ischemic myocardial late Gadolinium Enhancement lesions in patients with type 2 diabetes. Cardiovasc Diabetol 2020;19:184. 10.1186/s12933-020-01160-y 33092588 PMC7583253

[18] Gulsin GS, Henson J, Brady EM, et al. Cardiovascular determinants of aerobic exercise capacity in adults with type 2 diabetes. Diabetes Care 2020;43:2248–56. 10.2337/dc20-0706 32680830 PMC7440912

[19] Taqueti VR, Solomon SD, Shah AM, et al. Coronary Microvascular dysfunction and future risk of heart failure with preserved ejection fraction. Eur Heart J 2018;39:840–9. 10.1093/eurheartj/ehx721 29293969 PMC5939665

[20] Singh A, Jerosch-Herold M, Bekele S, et al. Determinants of exercise capacity and myocardial perfusion Reserve in asymptomatic patients with aortic stenosis. JACC Cardiovasc Imaging 2020;13:178–80. 10.1016/j.jcmg.2019.08.008 31542537

[21] Azevedo CF, Nigri M, Higuchi ML, et al. Prognostic significance of myocardial fibrosis Quantification by Histopathology and magnetic resonance imaging in patients with severe aortic valve disease. J Am Coll Cardiol 2010;56:278–87. 10.1016/j.jacc.2009.12.074 20633819

[22] Treibel TA, Kozor R, Schofield R, et al. Reverse myocardial remodeling following valve replacement in patients with aortic stenosis. J Am Coll Cardiol 2018;71:860–71. 10.1016/j.jacc.2017.12.035 29471937 PMC5821681

[23] Mahmod M, Francis JM, Pal N, et al. Myocardial perfusion and oxygenation are impaired during stress in severe aortic stenosis and correlate with impaired Energetics and Subclinical left ventricular dysfunction. J Cardiovasc Magn Reson 2014;16:29. 10.1186/1532-429X-16-29 24779370 PMC4009072

[24] Gulsin GS, Swarbrick DJ, Athithan L, et al. Effects of low-energy diet or exercise on cardiovascular function in working-age adults with type 2 diabetes: A prospective. Diabetes Care 2020;43:1300–10. 10.2337/dc20-0129 32220917

[25] Călin A, Roşca M, Beladan CC, et al. The left ventricle in aortic stenosis – imaging assessment and clinical implications. Cardiovasc Ultrasound 2015;13:22. 10.1186/s12947-015-0017-4 25928763 PMC4425891

[26] ATS/ACCP statement on cardiopulmonary exercise testing. Am J Respir Crit Care Med 2003;167:211–77. 10.1164/rccm.167.2.211 12524257

[27] Dhoble A, Enriquez-Sarano M, Kopecky SL, et al. Cardiopulmonary responses to exercise and its utility in patients with aortic stenosis. Am J Cardiol 2014;113:1711–6. 10.1016/j.amjcard.2014.02.027 24698467

[28] Nesti L, Pugliese NR, Sciuto P, et al. Type 2 diabetes and reduced exercise tolerance: a review of the literature through an integrated physiology approach. Cardiovasc Diabetol 2020;19:134. 10.1186/s12933-020-01109-1 32891175 PMC7487838

[29] Bilak JM, Gulsin GS, McCann GP. Cardiovascular and systemic determinants of exercise capacity in people with type 2 diabetes mellitus. Ther Adv Endocrinol Metab 2021;12:2042018820980235. 10.1177/2042018820980235 33552463 PMC7844448

[30] Musa TA, Treibel TA, Vassiliou VS, et al. Myocardial scar and mortality in severe aortic stenosis. Circulation 2018;138:1935–47. 10.1161/CIRCULATIONAHA.117.032839 30002099 PMC6221382

[31] Minamino-Muta E, Kato T, Morimoto T, et al. Causes of death in patients with severe aortic stenosis: an observational study. Sci Rep 2017;7:14723. 10.1038/s41598-017-15316-6 29116212 PMC5676690

