# Supplementary material for: Impact of diabetes on remodelling, microvascular function and exercise capacity in aortic stenosis
Source: Open Heart. 2023 Aug 16;10(2):e002441. doi: 10.1136/openhrt-2023-002441 (PMC10432628; doi:10.1136/openhrt-2023-002441)
Supplement: Supplementary data [file openhrt-2023-002441supp001.pdf]

Supplementary Material

Contents

Supplementary Table 1: List of biomarkers assessed 2

Supplementary Method: Image analysis 3

Supplementary Method: Plasma Biomarkers 3

Supplementary Figure 1: Number of participants with each investigation performed across the cohort. 4

Supplementary Table 2: Sensitivity analysis comparing aortic stenosis participants with and without diabetes, with the exclusion of participants with pre-diabetes. 5

Supplementary Table 3: Levels of plasma biomarkers in the two groups. 6

Supplementary Table 4: Proportion of each group with high levels of plasma biomarkers 7

Supplementary Table 5: Cox regression models for the primary composite endpoint 8

Supplementary Table 1: List of biomarkers assessed

Adiponectin
Angiopoietin-2
Chitinase-3-Like-1
C-reactive protein
Cystatin-C
Endoglin
Endostatin
Endothelin-1
Fatty acid binding protein-3
Fatty acid binding protein-4
Fas
Fibroblast growth factor-21
Fibroblast growth factor-23
Galectin-3
Growth differentiation factor-15
Intercellular adhesion molecule-1
Interleukin-1 beta
Interleukin-6
Interleukin-8
Interleukin-10
Lipocalin-2 (NGAL)
Matrix metalloproteinase-2
Matrix metalloproteinase-3
Matrix metalloproteinase-7
Matrix metalloproteinase-8
Matrix metalloproteinase-9
Matrix metalloproteinase-12
Myeloperoxidase
NTproANP
Osteoprotegerin
Osteopontin
Pentraxin-3
P-Selectin
proBNP
Renin
Serpin-E1
Suppression of tumorigenicity-2
Syndecan-1
Syndecan-4
Tenascin C
T-cell immunoglobulin and mucin domain 1
Tissue inhibitor of metalloproteinases-1
Tissue inhibitor of metalloproteinases-4
Tumour necrosis factor-alpha
Tumour necrosis factor-receptor 1
Tumour necrosis factor-receptor 2
Troponin T
Vascular endothelial growth factor
Vascular endothelial growth factor-receptor 1

Supplementary Method: Image analysis

LV volumes and mass were quantified using the built-in automated contouring tool using the short-axis stack with adjustments only made for clear and obvious errors. Biplane left atrial volumes were calculated using the 4- and 2-chamber cine images using the automated tool to contour throughout the cardiac cycle.

Tissue tracking was used to assess myocardial strain as previously described[1] to calculate global longitudinal strain (GLS) and global circumferential strain (GCS) as well as longitudinal and circumferential peak early diastolic strain rate (PEDSR). Systolic strain values are presented as absolute values such that lower values indicate worse myocardial mechanics[2]. Extracellular volume fraction (ECV) was calculated using pre- and post-contrast T1 maps and the haematocrit sampled on the same day as the CMR scan[3].

Perfusion images were first assessed qualitatively for regional perfusion defects by two experienced observers. Quantitative perfusion was assessed using a model independent deconvolution technique[4], or a dual-sequence gradient echo method with inline automated reconstruction and post-processing[5]. Myocardial perfusion reserve (MPR) was calculated as a ratio of global stress to rest myocardial blood flow. To minimise the impact of epicardial disease on assessment of coronary microvascular function, participants with infarction, regional perfusion defects or known obstructive coronary artery disease were excluded from quantitative perfusion analysis.

LGE images were qualitatively assessed by two experienced observers for focal fibrosis which was categorised as present or absent. Where present it was further categorised as infarction or non-ischemic. Right ventricular insertion point fibrosis was not classed as pathological.

Supplementary Method: Plasma Biomarkers

Plasma biomarkers for which >80% of participants had values below the lower limit of detection were excluded from analysis. Plasma biomarkers where 10-80% of values were at the lower limit of detection were dichotomised by first removing the values at the lower limit of detection. The Log10 mean was then used as the threshold for the remaining values. Values below the lower limit of detection or below the Log10 mean were classed as “Low”, whilst those above the Log10 mean were classed as “High”. Biomarkers where <10% met the lower limit of detection were treated as continuous variables with data Log10 transformed prior to group comparison.

Supplementary Figure 1: Number of participants with each investigation performed across the cohort.

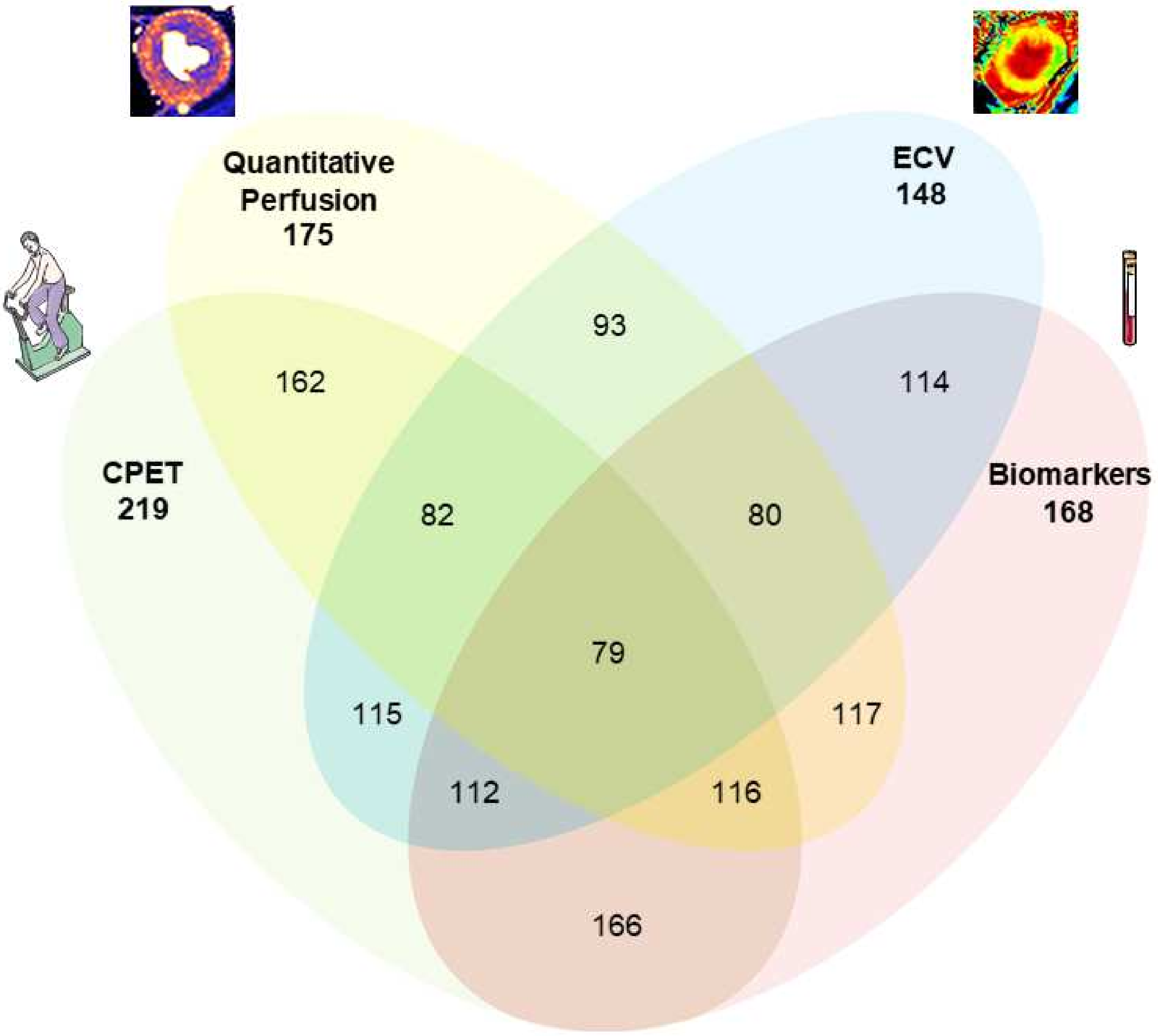

Supplementary Table 2: Sensitivity analysis comparing aortic stenosis participants with and without diabetes, with the exclusion of participants with pre-diabetes.

	Diabetes (n= 56)	Non-diabetes (n=171)	P value
**Age (years)**	70 (63 - 75)	68 (60-75)	0.296
**Sex, n (%) male**	44 (79)	130 (76)	0.696[†]
**Body mass index (kg/m^2)**	30.1 ± 5.2	28.1 ± 4.2	0.005
**HbA1c (%)**	6.8 ± 1.0	5.5 ± 0.3	<0.001
**Imaging***			
**LV EDVi (mL/m)**	88 ± 25	91 ± 21	0.238
**LV EF (%)**	68 ± 11	70 ± 7	0.122
**LVMi (g/m)**	97 ± 22	95 ± 24	0.613
**LVM/EDV (g/mL)**	1.14 ± 0.24	1.07 ± 0.22	0.177
**LV GCS (%)**	17.0 ± 3.4	18.2 ± 3.1	0.069
**LV GLS (%)**	14.0 ± 3.5	14.6 ± 2.8	0.569
**Presence of LGE, n (%)**	32 (59)	91 (54)	0.461[†]
**Native T1 (ms)**	1172 ± 83	1149 ± 73	0.983
**Extracellular volume (%)**	25.9 ± 3.1	24.9 ± 2.3	0.075
**Myocardial perfusion reserve**	2.02 ± 0.75	2.34 ± 0.68	0.048
**CPET [¥]**			
**Peak VO_2 (mL/kg/min)**	14.7 ± 4.9	17.5 ± 5.4	0.006
**Percentage predicted peak VO_2 (%)**	68 ± 21	76 ± 16	0.006
**Peak respiratory exchange ratio**	1.12 ± 0.16	1.10 ± 0.13	0.327

Ventricular volumes and mass were indexed to height. Predicted peak VO_2 calculated using the Wasserman/Hansen equation. Values presented as mean ± standard deviation, median (interquartile range) or n (%) as appropriate. Abbreviations: LV= left ventricle, EDVi = indexed end-diastolic volume, EF = ejection fraction, LVMi = indexed left ventricular mass, GCS =global circumferential strain, GLS = global longitudinal strain, LGE = late gadolinium enhancement. * = ANCOVA adjusted for age, sex, ethnicity, systolic BP, eGFR, BMI and aortic valve mean pressure gradient; [†] = chi-squared test; [¥] = ANCOVA adjusted for systolic BP, eGFR and aortic valve mean pressure gradient

Supplementary Table 3: Levels of plasma biomarkers in the two groups.

	Diabetes (n=32)	Non-diabetes (n=136)	Mean difference	95% CI Mean Difference		P value
Adiponectin	8.14 ± 1.08	8.17 ± 1.30	-0.024	-0.468	0.420	0.913
Angiopoietin-2	3.27 ± 0.63	3.13 ± 0.67	0.143	-0.114	0.401	0.273
Chitinase-3-Like-1	5.18 ± 0.88	5.08 ± 0.68	0.098	-0.181	0.376	0.490
Cystatin-C	6.68 ± 0.71	6.49 ± 0.72	0.197	-0.081	0.476	0.163
Endostatin	5.36 ± 0.50	5.19 ± 0.47	0.177	-0.008	0.362	0.060
Fatty acid binding protein-4	4.15 ± 0.73	3.99 ± 0.68	0.156	-0.111	0.423	0.251
Galectin-3	4.47 ± 1.03	4.38 ± 1.00	0.089	-0.300	0.478	0.651
hsTroponin I	0.83 ± 0.33	0.82 ± 0.48	0.006	-0.171	0.183	0.943
Lipocalin-2	5.58 ± 0.59	5.48 ± 0.68	0.102	-0.155	0.358	0.435
Matrix metalloproteinase-2	6.36 ± 0.83	6.14 ± 0.91	0.219	-0.128	0.567	0.214
Matrix metalloproteinase-3	4.51 ± 0.67	4.51 ± 0.76	0.004	-0.284	0.292	0.979
Matrix metalloproteinase-7	3.00 ± 0.55	2.78 ± 0.55	0.217	0.004	0.431	**0.046**
Matrix metalloproteinase-9	5.01 ± 0.48	4.92 ± 0.85	0.097	-0.210	0.404	0.534
Myeloperoxidase	5.78 ± 0.98	5.30 ± 1.55	0.486	-0.080	1.052	0.092
NTProANP	4.17 ± 1.14	3.97 ± 1.00	0.204	-0.196	0.604	0.315
NTproBNP	1.98 ± 0.64	1.73 ± 0.84	0.252	-0.022	0.526	0.071
Osteoprotegerin	1.34 ± 0.68	1.19 ± 0.77	0.155	-0.139	0.448	0.300
Osteopontin	4.14 ± 0.62	4.21 ± 0.52	-0.061	-0.270	0.148	0.563
P-Selectin	4.89 ± 0.96	4.91 ± 0.82	-0.021	-0.352	0.309	0.898
proBNP	2.53 ± 0.67	2.51 ± 0.77	0.024	-0.269	0.317	0.870
Renin	3.27 ± 0.59	2.82 ± 0.69	0.454	0.194	0.714	**<0.001**
Serpin-E1	5.05 ± 0.37	4.96 ± 0.72	0.091	-0.169	0.351	0.492
Syndecan-1	2.35 ± 0.64	2.26 ± 0.51	0.093	-0.115	0.300	0.379
Tenascin-C	4.31 ± 0.88	4.36 ± 0.59	-0.045	-0.300	0.210	0.729
Tissue inhibitor of metalloproteinases-1	5.56 ± 0.34	5.51 ± 0.38	0.051	-0.092	0.195	0.480

Values are Log_{10} transformed and presented as mean ± standard deviation

Supplementary Table 4: Proportion of each group with high levels of plasma biomarkers

	Diabetes (n=32)	Non-diabetes (n=136)	P value
C reactive protein	22 (69)	98 (72)	0.709
Endoglin	12 (38)	58 (43)	0.595
Endothelin-1	6 (19)	33 (24)	0.506
Fatty acid binding protein -3	1 (3)	15 (11)	0.171
Fas	15 (47)	35 (48)	0.223
Fibroblast growth factor -21	10 (31)	53 (39)	0.417
Fibroblast growth factor -23	9 (28)	26 (19)	0.259
Growth differentiation factor -15	19 (59)	50 (37)	**0.019**
Intercellular adhesion molecule -1	28 (88)	108 (79)	0.294
Interleukin-10	5 (16)	32 (24)	0.332
Interleukin-6	8 (25)	37 (27)	0.800
Interleukin-8	3 (9)	30 (22)	0.104
Matrix metalloproteinase-12	10 (31)	49 (36)	0.610
Matrix metalloproteinase-8	9 (28)	50 (37)	0.357
Pentraxin-C	10 (31)	49 (36)	0.610
Suppression of tumorigenicity-2	20 (63)	76 (56)	0.496
Syndecan-4	15 (47)	56 (41)	0.557
T-cell immunoglobulin and mucin domain -1	3 (9)	14 (10)	0.877
Tissue inhibitor of metalloproteinases -4	22 (68)	88 (65)	0.665
Tumour necrosis factor-alpha	8 (25)	32 (24)	0.861
Tumour necrosis factor-receptor 1	19 (59)	77 (57)	0.777
Tumour necrosis factor-receptor 2	18 (56)	46 (34)	**0.019**
Troponin T	7 (22)	29 (29)	0.438
Vascular endothelial growth factor-receptor 1	9 (28)	41 (30)	0.822
Vascular endothelial growth factor	3 (9)	25 (18)	0.219

Values represent number (percentage) with high levels

Supplementary Table 5: Cox regression models for the primary composite endpoint

	Primary composite endpoint									
	Baseline model (n=204)		Baseline + Renin (n=168)		Baseline + MMP7 (n=168)		Baseline + GDF-15 (n=168)		Baseline + TNF-R2 (n=168)	
	HR (95% CI)	P value	HR (95% CI)	P value	HR (95% CI)	P value	HR (95% CI)	P value	HR (95% CI)	P value
**Age**	**1.09 (1.05-1.12)**	**<0.001**	**1.08 (1.05-1.12)**	**<0.001**	**1.08 (1.05-1.12)**	**<0.001**	**1.08 (1.04-1.12)**	**<0.001**	**1.08 (1.04-1.12)**	**<0.001**
**Diabetes**	1.34 (0.76-2.37)	0.316	1.51 (0.77-2.96)	0.234	1.48 (0.77-2.85)	0.240	1.44 (0.75-2.78)	0.279	1.43 (0.73-2.78)	0.295
**AV mean PG**	0.98 (0.97-1.00)	0.089	0.99 (0.96-1.01)	0.334	0.99 (0.96-1.01)	0.262	0.99 (0.96-1.01)	0.361	0.99 (0.96-1.01)	0.363
**AVR**	**2.45 (1.09-5.51)**	**0.031**	2.16 (0.95-4.96)	0.068	2.17 (0.95-4.96)	0.065	2.15 (0.94-4.93)	0.072	2.16 (0.93-5.03)	0.073
**Renin**	-	-	0.87 (0.54-1.41)	0.566	-	-	-	-	-	-
**MMP-7**	-	-	-	-	0.79 (0.48-1.28)	0.334	-	-	-	-
**GDF-15**	-	-	-	-	-	-	0.97 (0.53-1.77)	0.911	-	-
**TNF-R2**	-	-	-	-	-	-	-	-	1.01 (0.55-1.88)	0.970

Abbreviations: AV = aortic valve, PG = pressure gradient, AVR = aortic valve replacement, MMP-7 = matrix metalloproteinase-7, GDF-15 = growth differentiation factor-15, TNF-R2 = tumour necrosis factor-receptor 2, NA = not applicable

1 Gulsin GS, Henson J, Brady EM, et al. Cardiovascular Determinants of Aerobic Exercise Capacity in Adults With Type 2 Diabetes. *Diabetes Care* 2020;**43**:2248-56.

2 Flachskampf FA, Biering-Sørensen T, Solomon SD, et al. Cardiac Imaging to Evaluate Left Ventricular Diastolic Function. *JACC: Cardiovascular Imaging* 2015;**8**:1071-93.

3 Haaf P, Garg P, Messroghli DR, et al. Cardiac T1 Mapping and Extracellular Volume (ECV) in clinical practice: a comprehensive review. *Journal of Cardiovascular Magnetic Resonance* 2016;**18**:89.

4 Steadman CD, Jerosch-Herold M, Grundy B, et al. Determinants and functional significance of myocardial perfusion reserve in severe aortic stenosis. *JACC Cardiovasc Imaging* 2012;**5**:182-9.

5 Kellman P, Hansen MS, Nielles-Vallespin S, et al. Myocardial perfusion cardiovascular magnetic resonance: optimized dual sequence and reconstruction for quantification. *Journal of Cardiovascular Magnetic Resonance* 2017;**19**:43.
